# Supplementary material for: Synergistic Effect of Perampanel and Temozolomide in Human Glioma Cell Lines
Source: J Pers Med. 2021 May 10;11(5):390. doi: 10.3390/jpm11050390 (PMC8150827; doi:10.3390/jpm11050390)

Supplementary Figure 1. Evaluation of necrosis (panel A) and live cells (panel B) in perampanel-treated glioblastoma cell lines. U87, U138, A172 and SW1783 glioma cell lines were cultured in complete medium containing perampanel 250 µM and 100 µM, approximately corresponding to IC50 and IC20, respectively. Cells were harvested after 24 h (light gray) and 48 h (dark gray) and apoptosis was measured by Annexin V-binding assay. The results are expressed as percent of apoptotic cells. P values were calculated by the two-side Student’s t test.

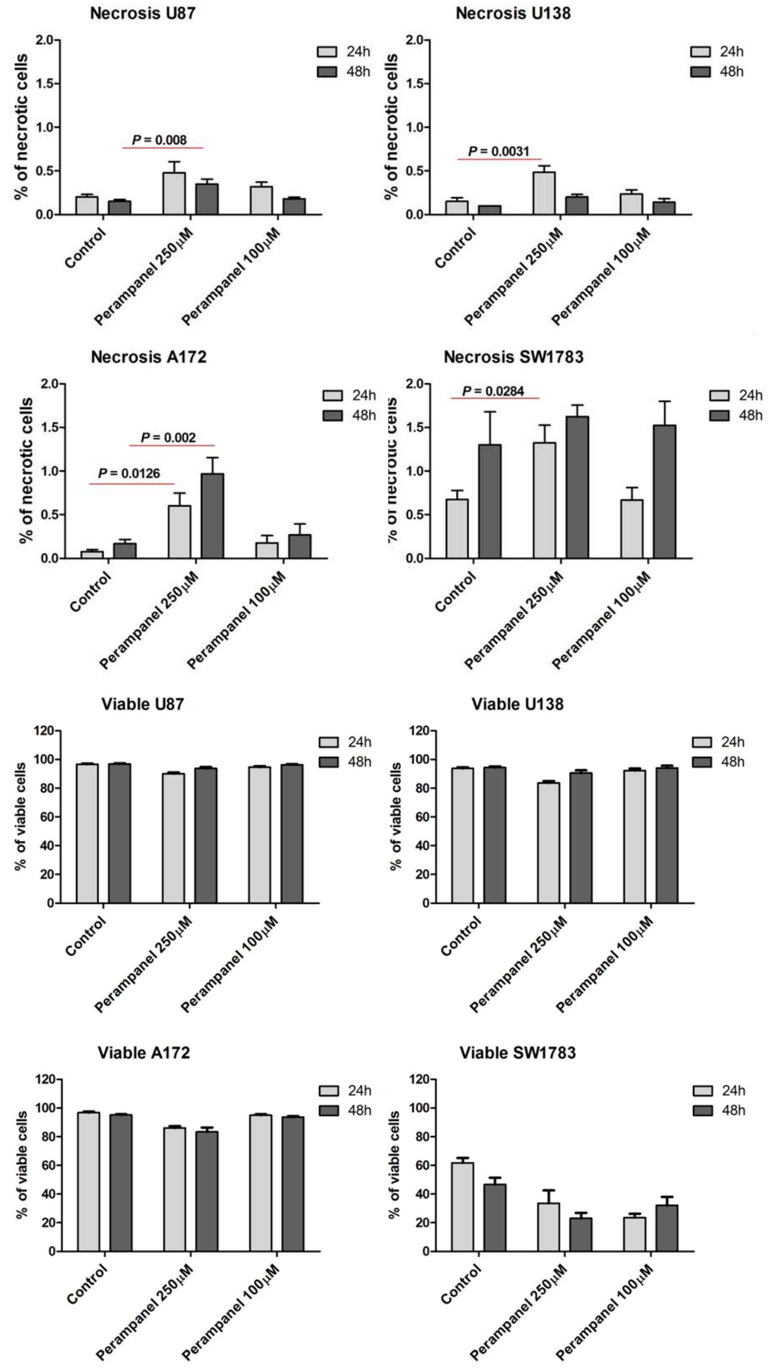

Supplement: Supplementary file 1 [file jpm-11-00390-s001.zip › jpm-1200488-supplementary.pdf]
